# Influence of Prosthetic Substrate, Cement, and Opaquer Liner on Color Matching of Translucent Zirconia- and Lithium-Based Ceramics

**DOI:** 10.3390/ma19071451

**Published:** 2026-04-05

**Authors:** Beata Dejak, Bartłomiej Konieczny, Agata Szczesio-Wlodarczyk, Wioleta Stopa

**Affiliations:** 1 Department of Prosthodontics, Medical University of Lodz, Pomorska 251, 92-213 Lodz, Poland; 2 University Laboratory of Materials Research, Medical University of Lodz, Pomorska 251, 92-213 Lodz, Poland; bartlomiej.konieczny@umed.lodz.pl (B.K.); agata.szczesio-wlodarczyk@umed.lodz.pl (A.S.-W.); wioleta.stopa@umed.lodz.pl (W.S.)

**Keywords:** translucent zirconia, lithium disilicate, zirconia-reinforced lithium silicate, color matching, translucency, opaquer liner, prosthetic substrate, resin cement, CAD/CAM, esthetic dentistry, all-ceramic crowns

## Abstract

**Highlights:**

- Optical properties of translucent zirconia- and lithium-based ceramics were evaluated.
- The translucency of the tested ceramics was as follows: LDS >> ZLS > zirconia ceramics.
- The use of an opaque liner reduced the translucency of the ceramics but did not mask the metal substrate.
- Transparent or colored luting material on tooth abutments provided the most favorable shade matching.
- Translucent zirconia restorations placed on metallic substrates resulted in unacceptable gray discoloration.
- The use of opaque luting material resulted in a color change and a lack of shading of the crowns.

**Abstract:**

The aim of this study was to evaluate the influence of prosthetic substrate type, resin cement shade, and opaquer liner application on the translucency and color matching of translucent zirconia- and lithium-based ceramics. Four A2-shade zirconia materials (Katana HTML Plus, STML, UTML, and YML), with and without an opaquer liner, lithium disilicate ceramics (Amber Mill LT and HT), and zirconia-reinforced lithium silicate (Celtra Duo) were investigated. Monolithic crowns and standardized rectangular specimens were fabricated using CAD/CAM technology and cemented with neutral, warm-shade, and opaque try-in pastes onto A2-shade composite resin and cobalt–chromium substrates. Color measurements were performed using a digital colorimeter based on the CIE L*a*b* system. Translucency parameters (TPs) and color differences (ΔE) relative to the A2 reference shade were calculated. Lithium-based ceramics exhibited significantly higher translucency than zirconia materials. Application of the opaquer liner on intaglio surface of crowns reduced their translucency. On A2-shade substrates, translucent zirconia luted with neutral or warm-shade paste demonstrated the most favorable color compatibility. In contrast, opaque try-in paste resulted in clinically unacceptable color deviations and loss of optical depth. On metallic substrates, most materials exhibited pronounced gray discoloration and substantial color mismatch, particularly lithium disilicate ceramics. These findings indicate that ceramic type, substrate color, opaquer liner application, and resin cement shade significantly influence the optical performance and final color outcome of all-ceramic restorations.

## 1. Introduction

Conventional 3Y-TZP (3 mol% yttria-stabilized tetragonal zirconia polycrystal) ceramics consist predominantly of tetragonal ZrO_2_ grains stabilized with 3 mol% Y_2_O_3_ and containing approximately 0.25 wt% Al_2_O_3_ [1]. These materials are characterized by fine, birefringent grains with sizes ranging from 0.2 to 1 µm [2]. Crystallographic anisotropy, a high density of grain boundaries, and the presence of alumina contribute to significant light scattering, resulting in low translucency. Despite these optical limitations, 3Y-TZP demonstrates excellent mechanical performance and has largely replaced metal substructures in fixed prosthodontics. It exhibits high stiffness, with a Young’s modulus of approximately 210 GPa [3]; high hardness (approximately 1448 HV1) [4]; and flexural strength in the range of 1100–1400 MPa [5]. Owing to its opacity, conventional 3Y-TZP is primarily used as a framework material veneered with more translucent ceramics to achieve acceptable esthetics [6]. However, clinical complications such as chipping or delamination of the veneering ceramic from fixed dental prostheses have been frequently reported [7].

To overcome these limitations, translucent zirconia ceramics have been introduced for the fabrication of monolithic restorations. This approach allows for more conservative tooth preparation, simplified laboratory procedures, and elimination of veneer-related technical complications while maintaining high mechanical strength [8]. Translucent zirconia materials contain increased amounts of yttria (3–6 mol% yttria-partially stabilized zirconia; 3–6Y-PSZ) [9] and are typically sintered at higher temperatures, reaching up to 1550 °C. Their microstructure contains a substantially higher fraction of the cubic zirconia phase at room temperature. Unlike the tetragonal phase, the cubic phase is optically isotropic [10]. Cubic ZrO_2_ grains are larger (approximately 2–5 µm), which reduces light scattering at grain boundaries and enhances translucency. The reported translucency parameter (TP) for these materials is approximately 9.9 [11]. However, increasing the cubic phase content significantly diminishes the transformation toughening mechanism associated with the tetragonal-to-monoclinic phase transformation at crack tips. As a result, the flexural strength of highly translucent zirconia is reduced to approximately 644.73–879.42 MPa [12]. Hardness values (approximately 1350 HV1) remain comparable to those of conventional 3Y-TZP [13].

Kongkiatkamon classified translucent zirconia ceramics according to yttria content into 3Y-TZP (3 mol% yttria with reduced alumina content), 4Y-PSZ containing 4 mol% Y_2_O_3_, 5Y-PSZ with 5 mol% yttria, and 6Y-PSZ with 6 mol% yttria [14]. Additionally, multilayer ceramics (e.g., M4Y-PSZ) have been introduced, consisting of layers with identical chemical composition but different translucency levels. Furthermore, shaded multilayer zirconia ceramics combining layers with different compositions, strength, and translucency (e.g., M3Y–5Y) are available [2]. In such materials, the cervical region provides increased strength, whereas the incisal layers exhibit higher light transmission [15].

Translucent zirconia systems currently available on the market may be categorized as follows:

- 3Y-TZP with reduced Al_2_O_3_ content (approximately 0.05 wt%), such as Ceramill Zolid (Amann Girrbach, Homebush, NSW, Austria), Vita YZ HT (Vita Zahnfabrik, Bad Säckingen, Germany), and Katana ZR HT and HTML Plus (Kuraray Noritake, Tokyo, Japan). Katana HTML Plus is an M3Y-PSZ multilayer zirconia composed of differently shaded layers: 35% dentin layer, two transitional layers of 15% each, and 35% enamel layer. According to the manufacturer, its translucency is approximately 45%, and the flexural strength is 1150 MPa [16].
- 4Y-PSZ, containing both tetragonal and cubic zirconia phases, characterized by higher translucency but lower flexural strength compared with 3Y-TZP. Examples include Ceramill Zolid HT+, Vita YZ ST, and Katana ZR STML (Super Translucent Multilayered). Katana STML is a multilayer material with a color gradient. Its translucency is 49%, and flexural strength is 748 MPa [16].
- 5Y-PSZ, containing approximately 53% cubic phase, exhibits significantly increased translucency but approximately 50% lower mechanical strength compared with conventional 3Y-TZP [17]. Representative materials include Ceramill Zolid FX, Vita YZ XT, and Katana ZR UTML (Ultra Translucent Multilayered). M5Y-PSZ, with a reported translucency of 51% and flexural strength of 557 MPa [16].
- Multilayer systems combining different zirconia compositions, such as Katana ZR YML, which integrates 3Y-TZP in the cervical region, 4Y-PSZ in the middle region, and 5Y-PSZ in the incisal region.

The clinical indications of zirconia ceramics depend on their mechanical and optical properties. Conventional 3Y-TZP is primarily used for frameworks of single crowns and multi-unit fixed dental prostheses and requires veneering due to its limited translucency. Low-alumina 3Y-TZP materials are indicated for monolithic crowns and short-span bridges. 4Y-PSZ is recommended for full-contour anterior and posterior crowns as well as three-unit fixed dental prostheses. In contrast, 5Y-PSZ is mainly indicated for anterior crowns, inlays, onlays, and veneers due to its enhanced translucency and reduced mechanical strength. Single-unit crowns may be fabricated from all types of zirconia ceramics.

Another group of esthetic restorative materials includes lithium disilicate ceramics (LDS; e.g., Amber Mill, HASS Bio, Gangneung-si, Gangwon-do, Republic of Korea) and zirconia-reinforced lithium silicate ceramics (ZLS; e.g., Celtra Duo, Dentsply Sirona). Lithium disilicate ceramics consist of approximately 60 vol% crystalline phase embedded in a silica (SiO_2_) glass matrix, with the presence of lithium orthophosphate crystals. Zirconia-reinforced lithium silicate ceramics contain approximately 18 wt% lithium oxide and 10 wt% zirconium dioxide dispersed within a silica-based matrix. The Li_2_Si_2_O_5_ crystals form elongated, needle-like structures measuring approximately 0.5–4 µm, whereas lithium silicate crystals range from 0.5 to 0.7 µm in size. Compared with zirconia ceramics, these materials exhibit lower mechanical performance, with flexural strength values ranging from 250 to 400 MPa [18]. A systematic review by Vichi et al. reported translucency parameter (TP) values for lithium disilicate materials ranging from 16 to 19 [19]. Owing to their favorable optical properties, these ceramics are widely used for anterior and posterior crowns, inlays, onlays, and veneers.

Achieving accurate shade matching between ceramic restorations and natural dentition remains a major clinical challenge. The final color of a restoration is influenced by multiple factors, including abutment shade, resin cement color, ceramic composition, microstructure, and material thickness. Given the broad range of available ceramic systems with different translucency levels, clinicians often encounter difficulties when selecting appropriate materials for anterior restorations, particularly in cases involving discolored teeth or post-and-core reconstructions [14]. Some studies have reported that super-translucent and ultra-translucent zirconia crowns exhibit significantly higher translucency than lithium disilicate crowns, even at a maximum thickness of 1.5 mm [20]. In contrast, other investigations have demonstrated inferior optical performance of translucent zirconia compared with highly translucent lithium disilicate ceramics [21]. These discrepancies indicate the need for further evaluation of the optical behavior of monolithic anterior crowns fabricated from different ceramic systems under various substrate conditions.

Therefore, the aim of this study was to evaluate the influence of ceramic type, prosthetic substrate, resin cement shade, and opaquer liner application on color matching, expressed as color differences relative to the standard A2 shade. The translucency parameter (TP) of the tested materials was determined, and full-crown in vitro assessments were performed.

The null hypothesis assumed that the investigated factors would not significantly affect the translucency or final color of the ceramic restorations.

## 2. Materials and Methods

**Figure 1:**
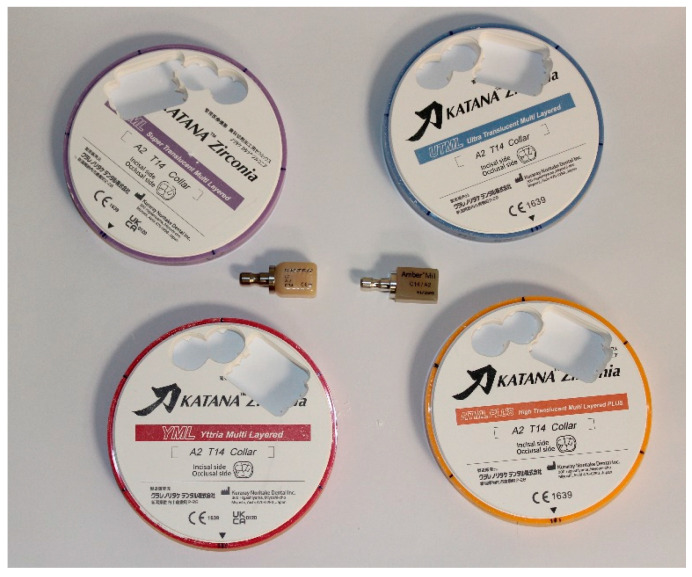
Ceramic material blocks used in the study for milling crowns and samples in the CAD/CAM system: zirconia blocks in shade A2—Katana HTML Plus, Katana STML, Katana UTML, and Katana YML (Kuraray Noritake Dental Inc., Tokyo, Japan); lithium disilicate Amber Mill (HASS Bio, Gangneung-si, Gangwon-do, Republic of Korea); and zirconia-reinforced lithium silicate Celtra Duo (Dentsply Sirona, Hanau, Germany).

Four blocks of translucent zirconia ceramics in shade A2 and thickness T14—Katana HTML, Katana STML, Katana UTML, and Katana YML (Kuraray Noritake Dental Inc., Tokyo, Japan)—as well as lithium disilicate LDS blocks Amber Mill LT and HT (HASS Bio, Gangneung-si, Gangwon-do, Republic of Korea) and zirconia-reinforced lithium silicate ZLS blocks Celtra Duo (Dentsply Sirona, Hanau, Germany) were used in this study (Figure 1). Katana zirconia blocks have a multilayer structure: 35% dentin layer, 30% transition layer, 35% enamel layer.

### 2.1. Crown-Based Experiments

#### 2.1.1. Abutment Fabrication

A KaVo tooth model No. 11 was prepared for an all-ceramic crown according to standard prosthetic guidelines: the preparation design included a 1.0 mm radial shoulder finish line, a total occlusal convergence angle of 6°, and a 2.0 mm incisal reduction. The prepared tooth was duplicated using a silicone impression material. A composite resin replica of the abutment was fabricated using Tetric Plus Flow A2 (Ivoclar Vivadent, Schaan, Liechtenstein). Subsequently, molten wax was poured into the silicone mold to obtain a wax pattern. The wax pattern was converted into a cobalt–chromium alloy (Co–Cr, Starbond COS, Scheftner, Mainz, Germany) using the lost-wax casting technique. This procedure resulted in composite abutments in shade A2 and a custom metal post-and-core with identical geometry.

#### 2.1.2. Crown Fabrication

Prosthetic crowns were fabricated using a CAD/CAM workflow from four translucent zirconia materials, lithium disilicate (LT and HT), and zirconia-reinforced lithium silicate. (Figure 1). The abutment mounted in a KaVo maxillary model was digitized using an Edge scanner (DOF Inc., Seoul, Republic of Korea). The STL files were imported into Exocad software (version 3.2, Darmstadt, Germany). The crowns were designed with identical dimensions and anatomy and had a labial thickness of 1 mm and a length of 11 mm. The space for cement was 0.08 mm. Due to the multilayer structure of the ceramics, the virtual crowns were centrally positioned within the blocks in Millbox software CIMsystem s.r.l. (Cinisello, Balsamo, Włochy, version 2022-04-15 (2021)), leaving a 1.5 mm margin at both the top and bottom. The data was sent to a CORiTEC Imes Icore 350i Loader Pro + CNC milling machine (Eiterfeled, Hesse, Germany), where the crowns were milled. Zirconia crowns were sintered in a Programat S1 1600 furnace (Ivoclar Vivadent, Schaan, Liechtenstein) according to the manufacturer’s recommendations. The sintering protocol for HTML Plus, UTML, STML, and YML materials consisted of heating at 10 °C/min to 1550 °C, holding for 2 h at the maximum temperature, followed by cooling at 10 °C/min to room temperature. A total of eight zirconia crowns were fabricated. Four crowns were infiltrated on the intaglio surface with an opaque liner (Opaque Liquid T1, Aidite, Qinhuangdao, China) prior to sintering, while the remaining four were left uncoated.

Additionally, crowns were milled from lithium disilicate (Amber Mill; HT and LT translucency) and zirconia-reinforced lithium silicate (Celtra Duo) blocks at a 1:1 scale. Lithium disilicate specimens underwent crystallization firing according to the manufacturer’s protocol to achieve the designated translucency level. All crowns were glazed.

#### 2.1.3. Cementation Procedure

Crowns were seated on composite and metal abutments in a KaVo maxilary model using three try-in pastes: transparent, tooth-color-shaded (Variolink Esthetic Try-In; shades: Neutral and Warm, Ivoclar Vivadent, Schaan, Liechtenstein), and opaque (Panavia V5 Try-In Paste, Kuraray Noritake Dental Inc., Okayama, Japan). The optical properties of the try-in pastes correspond to those of the respective resin cement shades provided by the manufacturers.

#### 2.1.4. Comparative Visual Assessment

Photographs of the crowns were obtained using a Nikon D5600 digital camera (Nikon Corporation, Tokyo, Japan) under standardized lighting conditions. The images were arranged into comparison maps. Each map included crowns fabricated from one ceramic type on different substrates (A2 composite and Co-Cr) and with different try-in pastes. Zirconia crowns were evaluated in two variants: with opaque liner; without opaque liner. The maps were independently evaluated by all authors. Color, lightness, and translucency of the ceramic crowns were assessed by comparing them with the A2 shade from the Vita Classical color guide (VITA Zahnfabrik, Bad Säckingen, Germany), as it is used by clinicians.

### 2.2. Color Measurement of Specimens

#### 2.2.1. Specimens Preparation

Rectangular specimens (1 mm × 11 mm × 20 mm) were fabricated using a CAD/CAM system (Exocad v3.2, Darmstadt, Germany; Imes Icore 350i Loader Pro Plus, Eiterfeld, Hesse, Germany) from four translucent zirconia blocks in shade A2 and thickness T14: Katana HTML Plus, STML, UTML, and YML (Kuraray Noritake Dental Inc., Tokyo, Japan). All specimens were positioned centrally within the blocks with a 1.5 mm margin. Two specimens were milled for each group: one coated on one side with an opaque liner (Opaque Liquid T1, Aidite, Qinhuangdao, China); one uncoated (control).

Additional specimens were prepared from lithium disilicate blocks (Amber Mill LT and HT, HASS Bio, Gangneung-si, Gangwon-do, Republic of Korea) and zirconia-reinforced lithium silicate blocks (Celtra Duo, Dentsply Sirona, Germany). These blocks were sectioned into 1.0 mm thick plates using a low-speed precision diamond saw (Tecmet 2000, Hitech Europe, Corsico, Italy). One surface of each specimen was glazed.

Ceramic specimens were evaluated independently and in combination with try-in pastes shades: Neutral and Warm (Variolink Esthetic Try-In, Ivoclar Vivadent; opaque paste Panavia V5, Kuraray Noritake Dental Inc.) on two substrates: A2 composite and Co-Cr metal. To ensure a standardized cement thickness of 0.08 mm, calibrated metal feeler gauges (TOYA S.A., Wrocław, Poland) were used.

#### 2.2.2. Colorimetric Measurements

Color measurements were performed using a digital colorimeter (ColorReader, Datacolor AG, Lawrenceville, NJ, USA) equipped with an integrated light source providing CIE D65 illumination and a 10° standard observer. This configuration ensured compliance with CIE Lab* measurement standards. The device was calibrated before each measurement using the supplied white reference standard. Each measurement was repeated three times and all readings were performed by a single operator. Due to the multilayer structure of the ceramics, a colorimeter positioning jig was fabricated in CAD/CAM system to ensure measurements were taken in the same place (at the center of each specimen).

#### 2.2.3. Translucency Parameter

All materials were measured against both black and white backgrounds (ColorChecker, Munsell Color, Grand Rapids, MI, USA). Three measurements were performed for each ceramic–substrate configuration. The translucency parameter (TP) was calculated based on color differences between mean values obtained on white and black backgrounds using the CIE Lab* formula:

(1) $$\Delta E=\sqrt{{∆L}^{2}+{∆a}^{2}+{∆b}^{2}}$$

where ΔL* = (L1 − L2), Δa* = (a1 − a2), and Δb* = (b1 − b2) (Formula (1)).

L* represents lightness (0 = black, 100 = white).

a* represents the green (−) to red (+) axis.

b* represents the blue (−) to yellow (+) axis.

#### 2.2.4. Masking Ability

Baseline measurements were first performed on an A2 composite substrate to establish reference color coordinates. Ceramic samples with three different test pastes were successively placed on an A2 composite substrate (Tetric EvoCeram, Ivoclar, Schaan, Liechtenstein) as a standard aesthetic reference background [22] or on a Co-Cr metal substrate (Starbond COS, Scheftner, Mainz, Germany), simulating a high-contrast metallic background. Masking ability was quantitatively assessed by calculating color differences (ΔE) in the CIE Lab* color space (Formula (1)) by comparing the color coordinates of the samples and the A2 reference background.

## 3. Results

### 3.1. In Vitro Crown Evaluation

**Figure 2:**
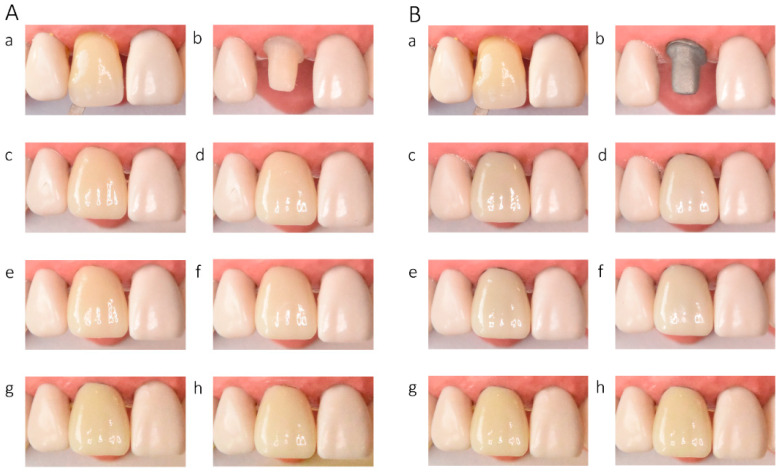
(**A**) Katana HTML Plus zirconia crowns on an A2-colored abutment; (**B**) Katana HTML Plus zirconia crowns a Co–Cr abutment: (**a**) Vita A2 shade tab; (**b**) A2 composite abutment; (**c**) crown cemented with neutral try-in paste; (**d**) crown with opaque liner cemented with neutral try-in paste; (**e**) crown cemented with A2 try-in paste; (**f**) crown with opaque liner cemented with A2 try-in paste; (**g**) crown cemented with opaque try-in paste; (**h**) crown with opaque liner cemented with opaque try-in paste.

**Figure 3:**
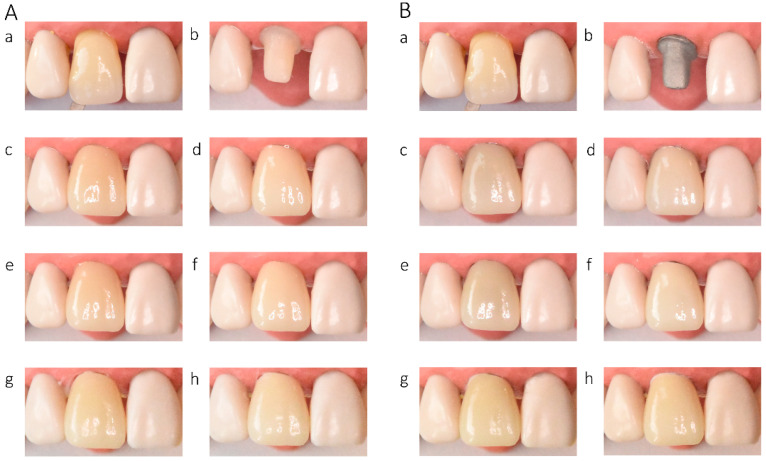
(**A**) Katana STML zirconia crowns on an A2-colored abutment; (**B**) Katana STML zirconia crowns on a Co–Cr abutment: (**a**) Vita A2 shade tab; (**b**) A2 composite abutment; (**c**) crown cemented with transparent try-in paste; (**d**) crown with opaque liner cemented with transparent try-in paste; (**e**) crown cemented with A2 try-in paste; (**f**) crown with opaque liner cemented with A2 try-in paste; (**g**) crown cemented with opaque try-in paste; (**h**) crown with opaque liner cemented with opaque try-in paste.

**Figure 4:**
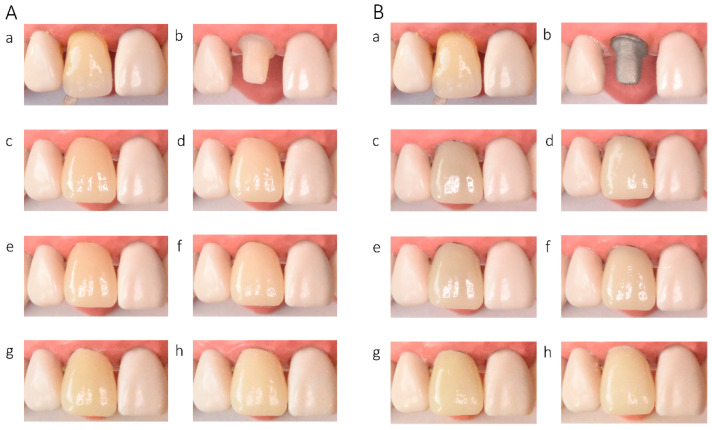
(**A**) Katana UTML zirconia crowns on an A2-colored abutment; (**B**) Katana UTML zirconia crowns on a Co–Cr abutment: (**a**) Vita A2 shade tab; (**b**) A2 composite abutment; (**c**) crown cemented with transparent try-in paste; (**d**) crown with opaque liner cemented with transparent try-in paste; (**e**) crown cemented with A2 try-in paste; (**f**) crown with opaque liner cemented with A2 try-in paste; (**g**) crown cemented with opaque try-in paste; (**h**) crown with opaque liner cemented with opaque try-in paste.

**Figure 5:**
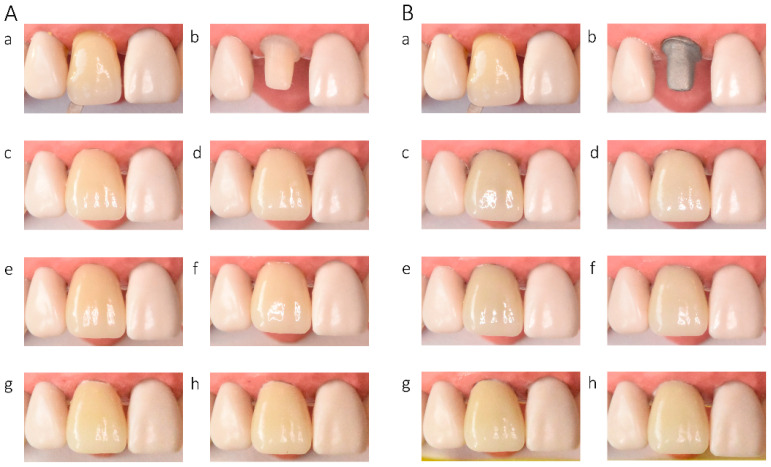
(**A**) Katana YML zirconia crowns on an A2-colored abutment; (**B**) Katana YML zirconia crowns on a Co–Cr abutment: (**a**) Vita A2 shade tab; (**b**) A2 composite abutment; (**c**) crown cemented with transparent try-in paste; (**d**) crown with opaque liner cemented with transparent try-in paste; (**e**) crown cemented with A2 try-in paste; (**f**) crown with opaque liner cemented with A2 try-in paste; (**g**) crown cemented with opaque try-in paste; (**h**) crown with opaque liner cemented with opaque try-in paste.

**Figure 6:**
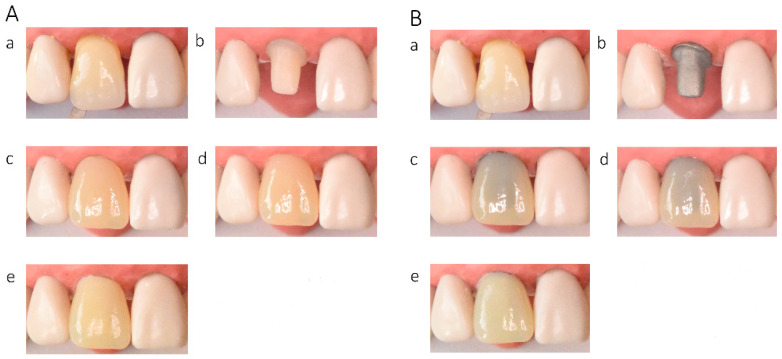
(**A**) Lithium disilicate Amber Mill LT crowns on an A2-colored abutment; (**B**) Amber Mill LT crowns on a Co–Cr abutment: (**a**) Vita A2 shade tab; (**b**) A2 composite abutment; (**c**) crown cemented with transparent try-in paste; (**d**) crown cemented with A2 try-in paste; (**e**) crown cemented with opaque try-in paste.

**Figure 7:**
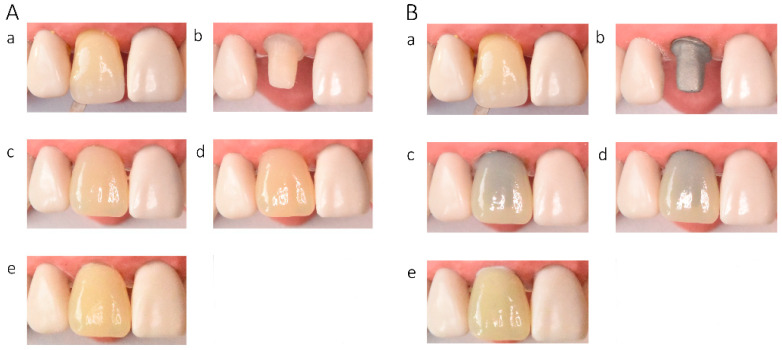
(**A**) Lithium disilicate (Amber Mill HT) crowns on an A2-colored abutment; (**B**) Amber Mill HT crowns on a Co–Cr abutment: (**a**) Vita A2 shade tab; (**b**) A2 composite abutment; (**c**) crown cemented with transparent try-in paste; (**d**) crown cemented with A2 try-in paste; (**e**) crown cemented with opaque try-in paste.

**Figure 8:**
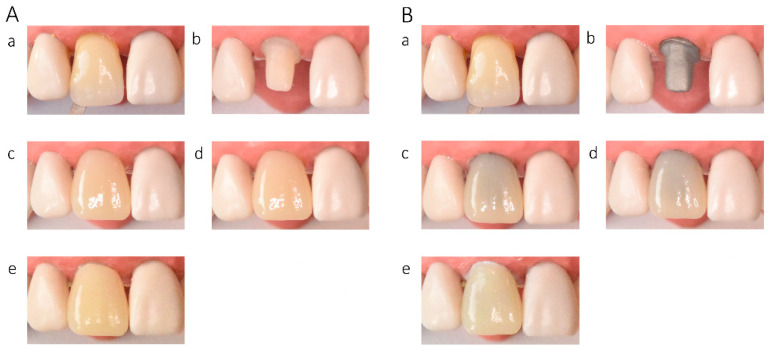
(**A**) Lithium silicate (Celtra Duo) crowns on an A2-colored abutment; (**B**) Lithium silicate (Celtra Duo) crowns on a Co-Cr abutment: (**a**) Vita A2 shade tab; (**b**) A2 composite abutment; (**c**) crown cemented with transparent try-in paste; (**d**) crown cemented with A2 try-in paste; (**e**) crown cemented with opaque try-in paste.

In the in vitro evaluation, crowns fabricated from lithium disilicate and zirconia-reinforced lithium silicate ceramics exhibited the highest translucency, followed by zirconia ceramics UTML > STML > HTML Plus > YML. Increasing ceramic translucency was associated with a darker perceived appearance of the crowns (Figure 2(Ac,Ae), Figure 3(Ac,Ae), Figure 4(Ac,Ae), Figure 5(Ac,Ae), Figure 6(Ac,Ae), Figure 7(Ac,Ae) and Figure 8(Ac,Ae)).

On A2-shaded tooth-colored abutments, crowns fabricated from translucent zirconia ceramics and cemented with transparent or warm-shaded Try-in paste exhibited a natural appearance and demonstrated close color matching with the A2 Vita Classical shade guide (VITA Zahnfabrik, Bad Säckingen, Germany) (Figure 2(Ac,Ae), Figure 3(Ac,Ae), Figure 4(Ac,Ae) and Figure 5(Ac,Ae)). The closest shade match was observed for STML and UTML ceramics cemented with warm-shaded paste (Figure 3(Ae) and Figure 4(Ae)). Application of an opaquer ceramic liner resulted in lightening of the crown color (Figure 2(Ad,Af), Figure 3(Ad,Af), Figure 4(Ad,Af) and Figure 5(Ad,Af)). In contrast, the use of opaque cement eliminated visible layering and reduced translucency, leading to an unfavorable esthetic outcome (Figure 2(Ag,Ah), Figure 3(Ag,Ah), Figure 4(Ag,Ah) and Figure 5(Ag,Ah)).

Zirconia crowns placed on custom metal posts and cores had a gray tint (Figure 2B, Figure 3B, Figure 4B and Figure 5B). The blue-gray tint of the metallic core was visible through the ceramic, especially after the application of a transparent or warm paste (Figure 2(Bc,Be), Figure 3(Bc,Be), Figure 4(Bc,Be) and Figure 5(Bc,Be)). The application of opaquer partially masked the substrate (Figure 2(Bd,Bf), Figure 3(Bd,Bf), Figure 4(Bd,Bf) and Figure 5(Bd,Bf)). The closest shade matching was achieved with STML crowns with opaquer and cemented with a warm-toned paste (Figure 3(Bd,Bf)). The opaquer paste also reduced the substrate discoloration; however, it resulted in a uniform and artificial appearance (Figure 2(Bg,Bh), Figure 3(Bg,Bh), Figure 4(Bg,Bh) and Figure 5(Bg,Bh)). Lithium disilicate crowns (Amber Mill) were characterized by significant translucency of the metallic core, resulting in a clinically unacceptable gray color (Figure 6B and Figure 7B). Lithium silicate (Celtra Duo) showed slightly better masking properties than lithium disilicate, although the restorations still had a grayish appearance (Figure 8B).

### 3.2. Colorimetric Analysis

**Figure 9:**
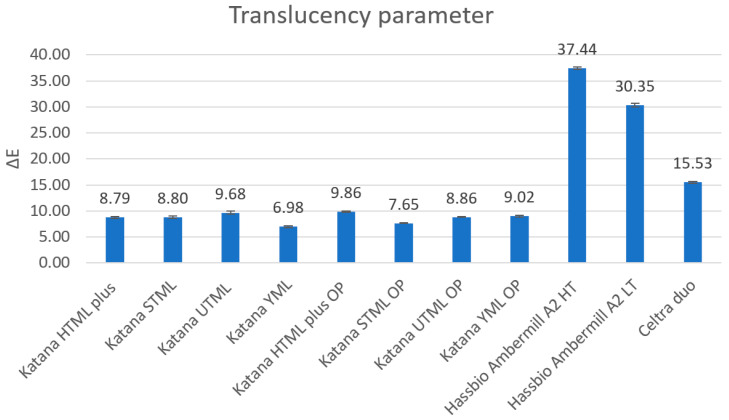
Mean values and standard deviations of the translucency parameter (TP) for the tested ceramic specimens, calculated from CIE Lab* color coordinates obtained over black and white backgrounds.

Colorimetric measurements performed on black and white substrates showed that lithium disilicate and zirconia-reinforced lithium silicate ceramics had the highest translucency. Translucency parameter (TP) values were 37.44 for Amber Mill HT, 30.35 for Amber Mill LT, and 15.53 for Celtra Duo. Zirconia ceramics were approximately three times less translucent than Amber Mill materials. HTML Plus and STML materials showed TP values of approximately 8.8, while UTML had the highest translucency (TP = 9.68) and YML the lowest (TP = 6.98) among the zirconia ceramics (Figure 9). The use of opaquer had little effect on the translucency parameters of this material.

**Figure 10:**
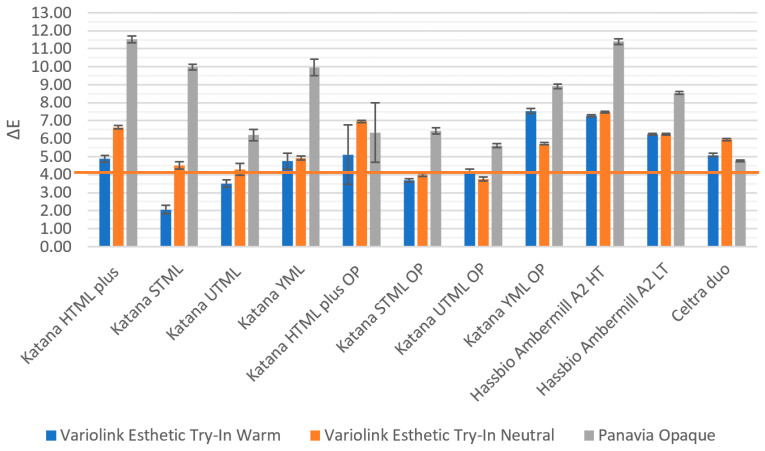
Mean values and standard deviations of color differences (ΔE) for the tested ceramic materials in A2 color, with different try-in pastes, placed on an A2 composite substrate, relative to the A2 reference shade Vitapan Classical color guide. The horizontal line indicates clinically acceptable difference.

On A2-colored substrates (Figure 10), the smallest color differences between the A2 reference and specimens cemented with warm shaded paste were observed for Katana STML (ΔE = 2.07) and UTML (ΔE = 3.50). The greatest deviations in specimens with color-cements from the A2 shade were recorded for YML (ΔE = 7.55) and Amber Mill HT (ΔE = 7.27). When specimens were cemented with neutral paste, the lowest color differences were observed for STML OP (ΔE = 3.98) and UTML OP (ΔE = 3.76). All remaining specimens differed from the A2 reference by ΔE > 4, with Amber Mill HT reaching ΔE = 7.47. Specimens cemented with opaque paste demonstrated the greatest deviations in color and translucency from the reference shade, with ΔE values exceeding 5. The best color performance with this cement was achieved by zirconia ceramics with an opaque liner (ΔE 5.61–6.44) and Celtra Duo (ΔE 4.77). The more translucent the ceramic, the more the opaque cement changed its color, with ΔE ranging from 8.55 to 11.53.

**Figure 11:**
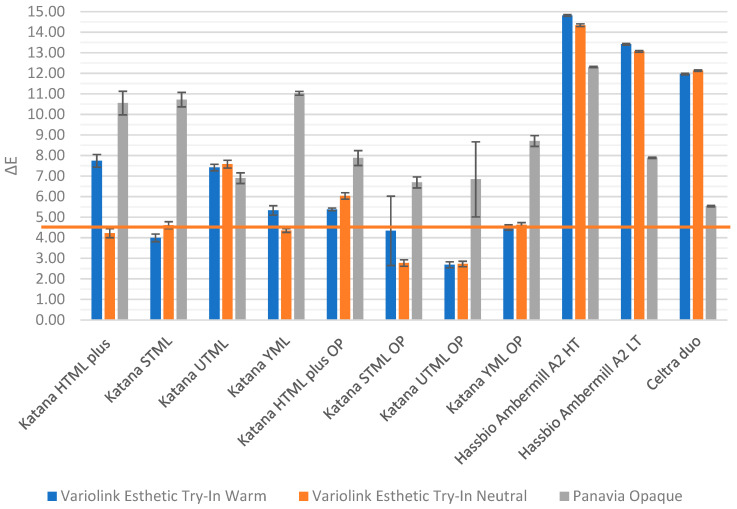
Mean values and standard deviations of color differences (ΔE) for the tested ceramic materials in A2 color, with different try-in pastes, placed on a cobalt–chromium (Co–Cr) substrate, relative to the A2 reference shade Vitapan Classical color guide. The horizontal line indicates clinically acceptable difference.

On metallic substrates (Figure 11), the color differences between most zirconia samples and the reference shade were significantly greater, exceeding ΔE > 4. Only STML OP and UTML OP ceramics with an opaquer liner, cemented with A2 or neutral cement, demonstrated relatively low deviations (ΔE = 2.7). The liner masked the background color. The ceramics became less translucent, but their color better matched the reference (Figure 11; Katana or Katana OP). The greatest color discrepancies were observed for lithium disilicate ceramics (ΔE = 13.07–14.81) and lithium silicate ceramics (ΔE ≈ 12) cemented with transparent cements. Application of opaque cement resulted in further color alteration of zirconia ceramics (ΔE = 5.61–11.53), but improved the color differences of lithium silicate ceramics, particularly Celtra Duo (ΔE = 5.54).

## 4. Discussion

The present study evaluated the translucency and masking ability of contemporary monolithic ceramic materials depending on substrate type, opaquer liner application, and cement shade.

The crown material has a significant impact on the aesthetics of restored teeth. Lithium disilicate ceramic (Amber Mill) demonstrated high translucency values (TP: LT 30.35; HT 37.44). This is consistent with reports by Vichy et al., who found that the TP of IPS emax CAD ceramic ranged from 12.64 to 37.3 [19]. Zirconia-reinforced lithium silicate (Celtra Duo) demonstrated an intermediate translucency (TP = 15.53), which corresponds to the value of 14.8 reported by Alayad et al. for 1 mm thick samples [23]. In contrast, zirconia ceramic demonstrated significantly lower translucency values (TP: 7–9.86). The TP values for Katana UTML were 9.68, which is consistent with the results of Fouda et al., who reported TP values of approximately 9.9 for zirconia UTML and STML [11]. The translucency of zirconia crowns is almost twice that of 18.7 for enamel and 16.4 for dentin [24]. The ceramic composition is the main factor determining translucency and significantly influences the final aesthetic outcome of restorations [11]. Lithium disilicate ceramics and zirconia-reinforced lithium silicate ceramics contain glassy matrices that increase light transmission [19], while polycrystalline zirconia exhibits increased scattering, thereby reducing translucency. New zirconia ceramics have a higher cubic phase content, which has larger and isotropic grains compared to the tetragonal phase [25]. This results in higher translucency for these materials.

In the present study, increased translucency of the ceramic (Amber > Celtra > Zircon Katana) was associated with a darker appearance of the crowns. This phenomenon can be explained by increased light transmission and background influences, which reduce the material’s intrinsic masking ability. Colorimetric testing revealed only minor differences between the zircon groups, but visual comparison of full-contour crowns revealed a noticeable advantage in translucency (HTML > STML > UTML > YML). Interestingly, lower-translucency materials generally demonstrated better shade matching to the A2 reference. This observation is consistent with the results of Haghi et al., who reported that more opaque ceramics may provide better color matching due to reduced background influence [26].

In colorimetric studies, differences in color and translucency between reference shades, natural teeth, and ceramic crowns are expressed as ΔE values. Currently, there is no universally accepted ΔE threshold for clinical imperceptibility. Ayash et al. suggested that a ΔE of 1.6 represents a clinically acceptable difference [27]. Douglas et al. reported that the 50% threshold for perceptibility among dentists was ΔE = 2.6, while differences exceeding ΔE = 5.5 were considered unacceptable [28]. Vichi et al. demonstrated that ΔE values below 1.0 were imperceptible to the human eye, values between 1.0 and 3.3 were perceptible but clinically acceptable, and values above 3.3 were perceptible to untrained observers [29]. In the present study, even ΔE differences of up to 4.5 were found to be clinically acceptable.

The results indicate that the prosthetic substrate is the most important factor influencing the color and aesthetics of translucent ceramic crowns. Crowns placed on A2-shade composite abutments demonstrated a favorable shade match with the reference material. However, these materials were ineffective in masking discolored or metallic substrates. Similar findings were reported by Miura et al., who demonstrated that abutment color significantly influenced the final shade of monolithic zirconia crowns placed on white and dentin substrates [30]. Kang et al. recommended limiting the use of translucent zirconia restorations to cases with natural-colored abutments and avoiding their use on discolored or bleached substrates [31]. Tabatabaian found that zirconia ceramics did not exhibit sufficient color masking ability to hide gray and black substrates. The ΔE values for the three groups—light gray, dark gray, and black—were 9.94 ± 2.11, 10.40 ± 2.09, and 13.34 ± 1.77 units, respectively [32]. Goveizi et al., however, reported that the mean ΔE*ab values for monolithic zirconia crowns remained below the detection threshold, regardless of the substrate type [33]. These results differ from the results of the present study, in which silver-colored metallic substrates significantly altered the color of monolithic zirconia crowns.

Applying an opaque liner to the intaglio surface of zirconia crowns (before sintering) partially masked the metal substrates, but a slight gray discoloration persisted. The liner reduced translucency and increased brightness. The most effective masking was observed for STML OP and YML OP ceramics. Kim et al. reported that stained liners can reduce the excessive translucency of lithium disilicate and zirconia ceramics by blocking background discoloration and improving natural color reproduction [34]. Soares et al. demonstrated that opaque stains applied to the intaglio surface provided excellent shade matching on A3, A4, and C2 substrates and acceptable matching on C4 substrates, but were ineffective in masking copper- and silver-colored metals [35].

Cement had a significant impact on the aesthetic outcome of translucent ceramic crowns. The use of tooth-colored or translucent try-in pastes on an uncolored abutment resulted in optimal, natural-looking restorations. On a metal abutment, these cements were completely ineffective, revealing a gray substructure. The use of opaque cements homogenized and flattened the restoration’s color, creating an artificial crown effect, regardless of the substrate. These results differ from those of Ayash et al., who reported that the shade of the resin cement did not significantly affect the final color perception [36]. Similarly, Haghi et al. found that nickel–chromium backgrounds could be masked with different cement shades (A2, opaque, white). However, they also noted that translucent zirconia and lithium disilicate restorations showed noticeable color changes after the use of opaque cement [26].

The null hypothesis was rejected. The type of ceramic, substrate color, and cement shade significantly influenced the translucency parameters and the final color outcome.

### Study Limitations

The main limitation of this study is the complexity of the research design, which included 33 experimental groups. Such a comprehensive comparison of multiple groups carries an inherent risk of type I errors, and maintaining high statistical power would require a significantly larger sample size. Therefore, these pilot results should be considered preliminary and require confirmation in future studies with larger cohorts.

To ensure measurement accuracy and avoid errors related to small field sizes, colorimetric analysis was performed on cubic samples rather than on the crowns themselves, which may not fully reflect the light behavior on complex anatomical surfaces.

Furthermore, although the study used standardized anatomical models, intraoral aging processes and the moist oral environment were not considered.

Try-in pastes were used instead of cements (due to the placement of multiple crowns with different colored bonding materials on the same abutments). The optical properties of both materials are very similar. The differences between cements and pastes lie in the setting method: cement contains reactive monomers that form a hard polymer. The test paste is a glycerin-based gel that does not harden and is completely water-soluble.

The parameter of variable ceramic thickness was not investigated in this article. A standardized tooth preparation was used and all crowns had a uniform veneer thickness of 1 mm. This may limit the clinical generalizability of the results, as thicker translucent ceramic layers reduce the translucency of monolithic restorations [37] and increase substrate masking [38].

Finally, it should be noted that due to the large surface area required for the experimental samples, achieving a completely uniform, opaque liner across the entire surface was impossible. This limitation could have affected the final optical results.

## 5. Conclusions

1. The type of zirconia-based ceramic material has a significant impact on the esthetic performance of restorations. Lithium disilicate exhibited the highest translucency, followed by zirconia-reinforced lithium silicate and translucent zirconia materials.
2. Application of an opaquer liner to ceramics reduces their translucency and can improve shade matching to the reference shade guide; however, it provides only limited masking of metal substrates.
3. The prosthetic substrate is the dominant factor influencing final crown color. Translucent ceramic crowns placed on tooth-colored abutments matched the reference color, while restorations supported on metal substrates exhibited unacceptable gray discoloration.
4. The shade of luting material influences the esthetics of all-ceramic crowns. Translucent zirconia crowns cemented with transparent or tooth-colored shade cements on tooth-colored substrates exhibited a natural appearance. The use of opaque cement resulted in loss of crowns’ translucency and elimination of visible layering effects.
5. Optimal esthetic outcomes with translucent ceramic restorations require careful coordination of material translucency, substrate color, and cement shade selection.

## Data Availability

The original contributions presented in this study are included in the article. Further inquiries can be directed to the corresponding author.
